# A 50-Year-Old Man With a History of Recurrent Exudative Right-Sided Pleural Effusion

**DOI:** 10.7759/cureus.26900

**Published:** 2022-07-15

**Authors:** Fortune O Alabi, Christopher O Alabi, Claudia Romero, Jenniffer Bates, Donald Elton

**Affiliations:** 1 Pulmonary, Critical Care and Sleep Medicine, Florida Lung, Asthma & Sleep Specialists, Orlando, USA; 2 Internal Medicine, Florida Lung, Asthma & Sleep Specialists, Orlando, USA; 3 Internal Medicine, HCA East Florida Westside/Northwest Medical Center, Plantation, USA; 4 Pulmonary Medicine, Florida Lung, Asthma & Sleep Specialists, Orlando, USA; 5 Pulmonary and Critical Care, Florida Lung, Asthma & Sleep Specialists, Orlando, USA

**Keywords:** pericardiectomy, septal bounce, recurrent pleural effusion, constrictive pericarditis, exudative pleural effusion

## Abstract

In this case report, we describe a 50-year-old man who presented to our facility for a second opinion after a year-long history of recurrent and now persistent right-sided exudative pleural effusion. On review of previous records, negative findings were seen in microbiological studies, including acid-fast bacilli, cytology, flow cytometry, and pleural biopsy using video-assisted thoracoscopy.

On transthoracic echocardiography performed during our evaluation, the expected respiratory variations across the mitral and tricuspid valves were not appreciated. This necessitated subsequent cardiac workup via magnetic resonance imaging, which showed a small pericardial fluid, thickened pericardium, and a septal bounce. The patient was surgically treated using a phrenic-to-phrenic pericardiectomy, following which his symptoms resolved completely.

Pleural effusions occur in approximately 40-60% of patients with constrictive pericarditis, and despite the known association of pleural effusions with constrictive pericarditis, the diagnosis of constrictive pericarditis is not readily entertained in patients with undiagnosed pleural effusions.

## Introduction

There are many causes of pleural effusions, and they range from benign conditions such as volume overload or pleurisy to malignant causes such as malignancy. The analysis of the fluid is imperative to ascertain the cause of the effusion. Light’s criteria are usually used to differentiate exudative effusions from transudative effusions [1]. The mechanism of both effusions is different and occasionally overlaps. Whereas exudative effusions are caused by disease processes that increase capillary permeability or impede lymphatic drainage, transudative effusions usually arise from conditions that increase hydrostatic pressure or decrease oncotic pressure. Medical conditions can present with either transudative or exudative effusions, such as pulmonary embolism [2], Meigs’ syndrome [3], and constrictive pericarditis [4]. In some cases, the diagnosis of the cause of the effusion might pose a diagnostic dilemma, such as in the case discussed in this report.

## Case presentation

A 50-year-old man with no significant medical history presented to our facility for a second opinion regarding recurrent right-sided pleural effusion and an indwelling chest tube that could not be removed secondary to persistent drainage of 250-300 mL daily.

The patient’s medical problem had started approximately one year previously when he presented to another hospital with severe chest pain and was diagnosed with idiopathic pericarditis. The chest computed tomography (CT) scan showed moderate pericardial effusion and a small right-sided pleural effusion. Transthoracic echocardiogram did not show evidence of cardiac tamponade. He was managed conservatively and discharged home to follow up with the cardiologist. The pericardial effusion gradually resolved over the next six months, but, unfortunately, the pleural effusion progressively increased. He was readmitted to the same hospital six months later with shortness of breath and underwent right thoracentesis with an aspiration of 1,500 mL of exudative fluid with lymphocytic pleocytosis and negative cytology. Flow cytometry and microbiological studies were negative. He underwent further therapeutic thoracenteses for recurrent pleural effusions with similar findings on fluid analysis.

He was admitted to the same hospital four weeks before this current admission for an elective right video-assisted thoracoscopic procedure. He underwent pleural biopsy, decortication, and mechanical pleurodesis, and a right-sided chest tube was placed intraoperatively. There was persistent drainage of pleural fluid from the chest tube, which made discontinuation of the chest tube difficult, and hence a second opinion was needed. The patient worked as a chef and had no history of smoking. He disclosed shortness of breath with activity but did not experience cough, fever, weight loss, or night sweats. There were no contributory clinical symptoms on a review of other systems.

On evaluation after admission, the patient appeared comfortable at rest and was not in any distress with a chest tube in place connected to a dry suction water seal chest drain. His vital signs on admission were as follows: axillary temperature of 98°F, pulse of 87 beats/minute, respiratory rate of 18 breaths/minute, blood pressure of 100/68 mmHg, and oxygen saturation of 98% on ambient air. Physical examination of his chest revealed decreased breath sounds and dull percussion notes on the basal right side but not on the left side. Cardiac examination revealed a regular rhythm and normal heart sounds without any murmurs, rubs, or gallops on auscultation. The Kussmaul sign was positive, but the pulsus paradoxus was negative. Musculoskeletal examination revealed bilateral pitting pedal edema up to his knees. The remainder of the physical examination revealed no further symptoms.

**Figure 1:**
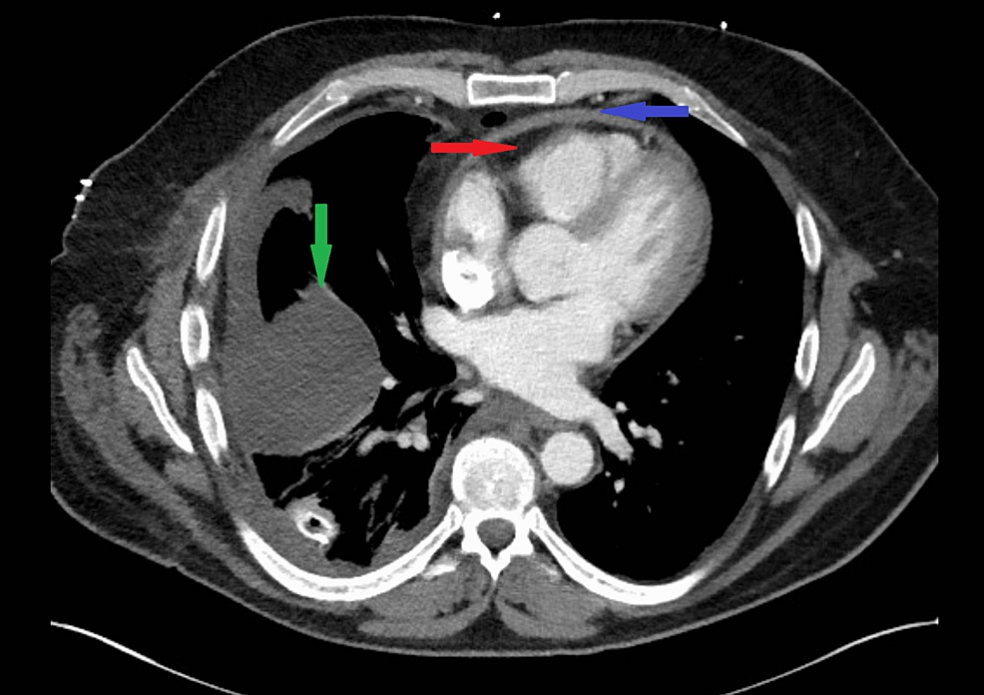
Chest computed tomography image showing pericardial thickening (blue arrow), small pericardial effusion (red arrow), and right-sided pleural effusion (green arrow).

Laboratory data at the time of admission revealed a white cell count of 9,000/mm^3^ with a normal differential. He had a hemoglobin level of 9 g/dL, a hematocrit of 40.4%, and a platelet count of 310,000/mm^3^. The basic metabolic profile (BMP) and N-terminal pro-brain natriuretic peptide were normal. Of note, tests for anti-nuclear antibody, rheumatoid factor, double-stranded DNA, Anti-neutrophil cytoplasmic antibody, Quantiferon TB Gold, and D-dimer were all negative. Chest X-ray showed right lung pleural effusion, while chest CT without contrast demonstrated pericardial thickening with trace pericardial effusion and loculated right pleural effusion that was larger than that during the previous examination 10 days prior (Figure 1). Pleural fluid was exudative by Light’s criteria, but no infection was seen on gram staining, acid-fast bacilli smear, and there was no growth from the cultures.

**Figure 2:**
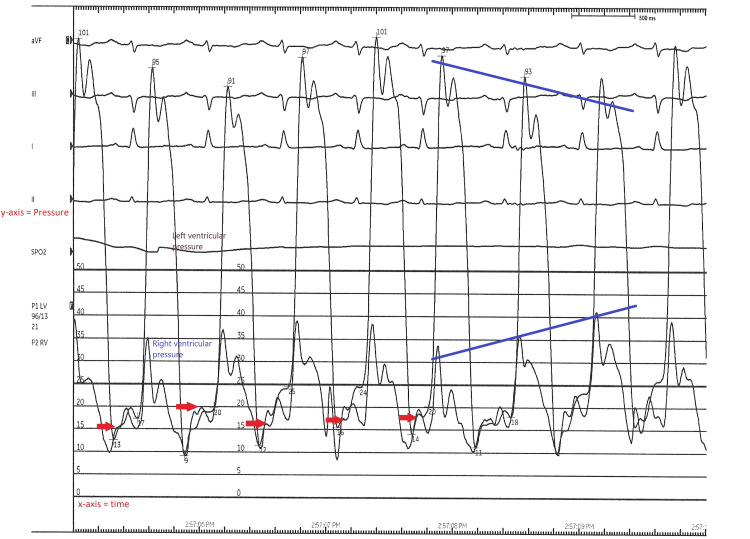
Cardiac catheterization tracings demonstrating respiratory discordance between the left and right ventricular pressures in the simultaneous measurement as indicated by the converging long blue lines. The red arrows indicate the classic constrictive pericarditis dip and plateau or “square root” sign.

Pleural biopsy results from recent surgery revealed reactive pleural tissue with nonspecific pleuritis without evidence of granulomas or malignancy. No infection was seen on gram staining of the tissue sample, and there was no growth from the culture. Transthoracic echocardiography/Doppler showed no pericardial effusion. There was evidence of mild biatrial enlargement with normal left ventricular size and function. The typical respiratory variations across the mitral and tricuspid valves with Doppler signals were not appreciated. Cardiac magnetic resonance imaging (MRI) (Video 1) showed thickened pericardium and septal bounce with a small volume of pericardial fluid, as well as other features, including biatrial enlargement and distention of the inferior vena cava (IVC) and hepatic veins. Cardiac catheterization (Figure 2) revealed mild luminal irregularities of the coronaries without any evidence of obstructive disease, normal left ventricular systolic function, mildly elevated right-sided pressure, and respiratory discordance in the simultaneous measurement of the right and left ventricular pressures. A clinical diagnosis of constrictive pericarditis was made based on the findings from the cardiac MRI and the result of the right and left heart catheterization.

**Video 1:** Cardiac magnetic resonance imaging showing a small volume of pericardial fluid, thickened pericardium, and septal bounce.

The patient subsequently underwent a phrenic-to-phrenic pericardiectomy on account of constrictive pericarditis complicated by recurrent right-sided exudative pleural effusion, peripheral edema, and other features consistent with heart failure. Pathologic examination of the excised pericardium revealed chronic nonspecific pleuritis. In view of the preceding diagnosis of acute idiopathic pericarditis with pericardial effusion, the etiology of the constrictive pericarditis was also considered to be idiopathic. His symptoms resolved completely after the pericardiectomy.

## Discussion

Constrictive pericarditis is a rare disease that is characterized by the loss of pericardial compliance leading to signs of right heart failure and reduced cardiac output. It can pose a diagnostic dilemma even to experienced physicians [5]. However, suspicion is raised when dealing with patients with signs of right heart failure, dyspnea with activity, bilateral lower extremity swelling, or unexplained pleural effusion. Constrictive pericarditis can arise from any process that affects the pericardium. The two leading causes of constrictive pericarditis in the developed world are cardiac surgery and idiopathic cases of pericarditis [6].

Pleural effusion may be seen in many conditions, and its presence may lead to chest pain or shortness of breath; however, usually, analysis of the fluid guides the diagnosis of the underlying disease process, such as in cases of malignancy or empyema.

Although pleural effusions are common in pericardial diseases [7], only a few cases require thoracentesis, thereby reducing the number of pleural fluid analyses that are performed on patients with pericardial diseases. Pleural effusions occur in approximately 40-60% of patients with constrictive pericarditis [8]. Despite the known association of pleural effusions with constrictive pericarditis, the diagnosis of constrictive pericarditis is not readily entertained in patients with undiagnosed pleural effusions [9]. Mechanistically, pleural fluids from constrictive pericarditis are usually transudative in nature secondary to elevated hydrostatic pressure with diastolic dysfunction of the left ventricle. Occasionally, transudative pleural fluid might arise from the transdiaphragmatic migration of ascitic fluid originating from elevated systemic venous pressure and posthepatic portal hypertension [10]. The elevated intra-atrial pressure alone is unlikely to explain the mechanism of pleural effusions in patients with constrictive pericarditis because the pressures are similar in those with and without pleural effusions. Therefore, it is unsurprising that the occurrence of exudative pleural effusion does not naturally trigger the differential diagnosis of constrictive pericarditis.

The mechanism of exudative pleural effusions is usually from increased capillary permeability, lymphatic obstruction, or transdiaphragmatic migration of exudative fluid from the peritoneal cavity [11]. The most common causes are infections, inflammatory processes, postcardiac injury syndrome, or malignancy. Other uncommon causes include pancreatitis, pulmonary embolism, radiation pleuritis, Meigs’ syndrome, asbestos-related pleural disease, and drug-induced pleural disease. The cause of exudative effusion in constrictive pericarditis is not well known, but possible mechanisms include pericardial inflammation, impairment of lymphatic drainage from elevated right atrial pressure, and the contribution of underlying disease processes responsible for constrictive pericarditis [4].

The pleural effusions in constrictive pericarditis and pleural diseases are generally left-sided, and, when bilateral, they are usually more on the left than the right. Most pleural effusions in constrictive pericarditis are exudative according to Light’s criteria, and some cases of transudative effusions have also been reported [12].

The most common causes of unexplained exudative pleural effusion are malignancy and tuberculosis. A closed needle biopsy can be performed for suspected tuberculosis-induced pleural effusion, especially when the pleural fluid is exudative and shows lymphocytic pleocytosis. We propose that minimal diagnostic testing should be performed to rule out constrictive pericarditis in patients with unexplained exudative pleural effusion before subjecting them to surgical procedures.

## Conclusions

Pleural effusions may be seen in many conditions, and their presence may lead to chest pain or shortness of breath, which is often the reason for presentation in the hospital. The most common causes of unexplained exudative pleural effusion are malignancy and tuberculosis. The diagnosis of constrictive pericarditis is not common in clinical practice, and its association with exudative effusion is not routinely considered. Unexplained pleural effusions can be the initial presentation manifestation in up to 30% of cases of constrictive pericarditis. Constrictive pericarditis should be considered in the differential diagnosis of unexplained or recurrent pleural effusion once infection and malignancy have been excluded.
